# Water stress intensified the relation of seed color with lignan content and seed yield components in flax (*Linum usitatissimum* L*.*)

**DOI:** 10.1038/s41598-021-02604-5

**Published:** 2021-12-14

**Authors:** Sara Zare, Aghafakhr Mirlohi, Ghodratollah Saeidi, Mohammad R. Sabzalian, Ehsan Ataii

**Affiliations:** Department of Agronomy and Plant Breeding, College of Agriculture, Isfahan University of Technology, 84156 83111 Isfahan, Iran

**Keywords:** Plant breeding, Plant genetics, Plant stress responses

## Abstract

This study aimed to investigate the effect of yellow and brown seed coat color of flax on lignan content, seed yield, and yield components under two contrasting environments of non-stress and water stress conditions. The water stress environment intensified the discrimination between the two seed color groups as the yellow seeded families had lower values for seed yield components under the water stress. Heritability and the genetic advance for seed yield were significantly higher in brown-seeded families than those of yellow-seeded ones at water stress conditions. Secoisolariciresinol diglucoside (SDG) as the chief lignan in flaxseed was more abundant in yellow-seeded families under the non-stress environment but under water stress conditions, it increased in brown seeded families and exceeded from yellow ones. Considering that the brown and yellow seed color families were full sibs and shared a similar genetic background but differed in seed color, it is concluded that a considerable interaction exists between the flax seed color and moisture stress concerning its effect on seed yield and yield components and also the seed SDG content. Brown-seeded genotypes are probably preferred for cultivation under water stress conditions for better exploitation of flax agronomic and nutritional potentials.

## Introduction

Flax (*Linum usitatissimum* L*.*) is an essential ancient crop with many uses worldwide^1^. Flaxseed contains 37–42% oil, classified as a dry oil due to high alpha-linolenic acid (ALA) content. In recent years with genetic modifications through mutation breeding, modified genotypes contain less than 5% linolenic acid in the oil suitable for edible purposes^2,3^. Flaxseed was found to be very beneficial for human health because of its omega-3 fatty acids (alpha-linolenic acid), lignans, and fiber^4^. The ratio of omega-3 to omega-6 fatty acids (1:0.3) in flaxseed is due to the high content of alpha-linolenic acid, which is superior to the other sources such as corn, soybeans, and fish oil^5,6^.

The main breeding goals of flaxseed generally include improving seed yield, seed oil content, oil quality, early ripening, and appropriate plant height. Yield is the most critical and complex trait in crop plants which is highly correlated with other characteristics^7^. The selection for seed yield is complicated because of its multi-genic nature and predominantly environmental dependence. Therefore, instead of direct selection, more efforts are devoted to yield components or traits that may indirectly affect seed yield^8–10^. In flax, seed yield can be improved by enhancing its components including the number of seeds per capsule, the number of capsules per plant, and thousand seed weight^7,8^.

Regular flaxseeds are usually characterized by two primary seed coat colors, brown and yellow (golden), and both contain greater than 50 percent ALA in the oil, making it unsuitable for cooking. To bypass the high oxidation instability of ALA, low linolenic-yellow seeded varieties with the trademark of Linola or Solin that contains less than five percent linolenic fatty acid in seed oil have been developed for edible purposes, using mutation breeding^11–13^. Flax brown seed color is controlled by the presence of at least one dominant allele in each of the G, B1, and D loci, while yellow seed color results from homozygous recessive alleles in at least one of these three loci^14,15^. Seed coat color in flax is reported to be associated with seed yield and some traits related to seed quality^11^. Using near-isogenic populations, Saeidi and Rowland^12^ found that seed color had an effect on flax seed vigor with yellow-seeded lines having lower seed vigor than brown-seeded lines. Several other reports also indicated that yellow-seeded flax had lower seed yield but higher oil concentration than brown-seeded lines. Later maturity, lower seed germination, and seed damage including the natural splitting of the seed coat and mechanical cracking are other factors associated with yellow seed color in flax^16–18^.

Considering that the previous reports mostly used brown and yellow seed color genotypes of flax having different genetic backgrounds under normal water conditions, our search was driven to test multiple families of full-sib progenies each pair sharing the same genetic background but differentiating in seed color under normal and water stress conditions. Moreover, tannins and lignans are considered potent secondary metabolites in flax. Tannins are present as pigments in seed coats, causing the brown color in flaxseed^19^. Both tannins and lignans have significant biological effects, but their impact on drought tolerance of flax has not been considered despite the presence of two major seed colors. Therefore, the present study aimed (1) to determine the response of multiple full-sibs flax genotypes segregating in brown and yellow seeds, obtained from intra-specific crosses to water stress and (2) to estimate the correlation of the two seed colors with economically important characteristics under the two water conditions.

## Results

The variance analysis for the traits in the two moisture environments is shown separately in Tables 1 and 2. Table 3 shows the analysis of stress tolerance indices, Tables 4 and 5 contain the calculation of genetic parameters, and Table 6 includes Chi-square (χ^2^) values.

### Univariate data analysis

#### Analysis of variance in the non-stress environment

The analysis of variance (Table 1) showed that the effect of F_3_ families was significant (*p* < 0.01) for all measured traits, and also the effect of parents was significant (*p* < 0.01) for all the traits except for seed yield.

The effect of families from direct crosses was significant for all measured traits, except for NSC in brown-seeded families and NC and NSC in yellow-seeded families, respectively. The effect of brown versus yellow-seeded families in direct crosses was also significant for DF, DC, DR, and TWS (Table 1).

Families' effect within reciprocal crosses (Table 1) was significant for all the traits except NSC in yellow-seeded families. The effect of brown versus yellow-seeded families in these crosses was significant only for DR, and there was no significant difference for the other traits (Table 1).

Based on the ANOVA results (Table 1), the effects of cytoplasmic replacement were also significant for most of the traits except for NC, CD, NSC, and seed yield, indicating considerable differences between direct and reciprocal crosses. Also, progenies versus parents' effects were significant in DF, DC, DR, NB, NSC, and seed yield (Table 1).

**Table 1:** Analysis of variance (ANOVA) for studied traits in flaxseed families under non-stress conditions.

S.O.V	df	Mean of squares									
		DF (day)	DC (day)	DR (day)	PH (cm)	NB (pp)	NC (pp)	CD (mm)	NSC (pp)	TWS (g)	Seed yield (g/m^2^)
Replication	2	167.34**	1.34^ns^	45.9**	92**	3.18	1153.7	2.65**	7.79**	0.111^ns^	714.9^ns^
Family	103	22.52**	18.16**	24.38**	217.57**	1.12**	226.9**	0.214**	0.69**	2.68**	4890**
Parent	7	58.3**	36.66**	27.47**	429.7**	3.04**	687.14**	0.461**	1.45**	7.91**	1652.7^ns^
Direct cross	47	19.41**	12.76**	28.48**	215.34**	1**	177.38**	0.26**	0.54^ns^	2.27**	4780.4**
Yellow	20	16.55**	12.43**	30.81**	93.66**	0.81**	119.94^ns^	0.26**	0.47^ns^	1.81**	6189.9**
Brown	26	22.11**	13.35**	27.36**	302.03**	1.18**	216.79**	0.27**	0.62^ns^	2.71**	3823.9**
Yellow vs. Brown	1	63.33**	23.42**	42.5**	2.26^ns^	0.4^ns^	37.86^ns^	0.18^ns^	0.47^ns^	6.4**	1000.6^ns^
Reciprocal cross	47	21.07**	21.5**	19.8**	196.39**	0.86**	155.46*	0.13**	0.72*	2.38**	4253.4**
Yellow	20	17.52**	21.28**	20.18**	176.7**	1.15**	166.01*	0.14**	0.67^ns^	2.19**	5624.1**
Brown	26	24.21**	22.22**	19.8**	215.74**	0.66**	148.7*	0.13**	0.77*	2.62**	2988.9**
Yellow vs. Brown	1	10.52^ns^	7.05^ns^	11.21**	87.05^ns^	0.41^ns^	120.07^ns^	0.008^ns^	0.45^ns^	0.01^ns^	3388.74^ns^
Direct vs. Reciprocal	1	205.03**	178.9**	37.55**	691.2**	2.36**	119.98^ns^	0.03^ns^	1.24^ns^	11.7**	642.5^ns^
Cross vs. Parent	1	23.24**	22.3**	23.4**	42.1^ns^	4.3**	16.75^ns^	0.08^ns^	2.01*	0.05^ns^	5970.1*
Error	206	3.003	2.18	0.66	24.98	0.29	90.85	0.062	0.44	0.093	1100.8
CV (%)		2.7	2.14	0.83	7.68	22.53	29.28	3.69	7.49	5.88	15.03

DF: Day to Flower. DC: Day to Capsule formation. DR: Day to Ripening. PH: Plant Height. NB: Number of Branches per plant. NC: Number of Capsules per plant. CD: Capsule Diameter. NSC: Number of Seeds per Capsule. TWS: Thousand Seed Weight. pp: per plant.

^ns^Non significant.

*Significant at 0.05 level of probability.

**Significant at 0.01 level of probability.

#### Analysis of variance in the stress environment

Under stress conditions, the effect of families was significant for all the traits, and also the effect of parents was significant for all traits except NB, NSC, and seed yield (Table 2). The effect of families within direct crosses was significant for all the studied traits except for NSC in yellow-seeded families and NC and NSC in brown-seeded families, respectively. The comparative effect of yellow versus brown-seeded families within crosses was significant (*p* < 0.01) for DF, DR, DC, CD, NSC, TWS, and seed yield (Table 2).

Families' effect within reciprocal crosses was significant for all the traits except for NSC in brown-seeded families. The effect of yellow versus brown-seeded progenies in reciprocal crosses showed significant differences in DR, CD, NSC, TWS, and seed yield. The effects of cytoplasmic replacement were also significant for all the traits except for NSC, and the effect of parents versus progenies was significant for most traits except for NC, NSC, and TWS (Table 2).

**Table 2:** Analysis of variance (ANOVA) for studied traits in flaxseed families under water stress conditions.

S.O.V	Df	Mean of squares									
		DF (day)	DC (day)	DR (day)	PH (cm)	NB (pp)	NC (pp)	CD (mm)	NSC (pp)	TWS (g)	Seed yield (g/m^2^)
Replication	2	4.93*	1.06^ns^	56.9**	41.39	0.24^ns^	468.8**	0.27*	1.07^ns^	0.028^ns^	222.2^ns^
Family	103	14.58**	8.12**	73.3**	141.8**	0.74**	31.13**	0.32**	0.79**	1.66**	3656**
Parent	7	23.78**	20**	35.97**	366.6**	0.45^ns^	41.38*	0.48**	0.94^ns^	3.69**	365.7^ns^
Direct cross	47	13.01**	5.04**	31.88**	120.4**	0.72**	29.31*	0.33**	0.78^ns^	1.69**	4221**
Yellow	20	12.81**	4.8**	33.14**	100.9**	0.56**	25.44*	0.37**	0.61^ns^	1.32**	4284**
Brown	26	13.4**	5.31**	32.08**	139.7**	0.76**	23.49^ns^	0.3**	0.83^ns^	2**	3000**
Yellow vs. Brown	1	14.92**	4.58*	71.78**	14.5^ns^	0.92^ns^	9.68^ns^	0.55**	3.44**	7.01**	19,754**
Reciprocal cross	47	15.26**	9.51**	13.4**	134.5**	0.77**	32.08**	0.29**	0.81*	1.39**	3697**
Yellow	20	15.51**	9.8**	13.07**	122.36**	0.84**	27.21*	0.27**	1.17**	1.44**	2959**
Brown	26	15.51**	9.55**	14.12**	149.01**	0.73**	36.26**	0.31**	0.47^ns^	1.38**	3257**
Yellow vs. Brown	1	3.73^ns^	1.94^ns^	1.38*	1.49^ns^	0.48^ns^	20.88^ns^	0.33*	2.24*	0.47*	29,874**
Direct vs. reciprocal	1	68.05**	65.17**	7.67**	519.52**	6.05**	56.7*	1.19**	0.25^ns^	4.98**	1930*
Cross vs. parent	1	49.38**	13.76**	52.89**	116.16**	2.54**	24.98^ns^	0.78**	0.43^ns^	0.001^ns^	21,161**
Error	206	1.57	0.92	0.32	21.43	0.29	15.46	0.08	0.49	0.09	482.15
CV (%)		2.01	1.42	0.65	8.46	20.13	20.79	4.37	10.01	6.8	17.35

DF: Day to Flower. DC: Day to Capsule formation. DR: Day to Ripening. PH: Plant Height. NB: Number of Branches per plant. NC: Number of Capsules per plant. CD: Capsule Diameter. NSC: Number of Seeds per Capsule. TWS: Thousand Seed Weight. pp: per plant.

^ns^Non significant.

*Significant at 0.05 level of probability.

**Significant at 0.01 level of probability.

#### Analysis of variance for tolerance indices

The results obtained from the variance analysis for stress tolerance indices showed that only the SSI index was significantly different between parents. For all calculated tolerance indices, significant differences were observed among families, direct crosses, reciprocal crosses, progenies versus parents, and yellow and brown-seeded families within the crosses. The only exception was the MP index, which did not show a significant difference between yellow versus brown-seeded progenies in reciprocal crosses (Table 3).

Analysis of variance (Table 3) showed that the effects of cytoplasmic replacement were significant only for the TOL index. According to the TOL index, parents, yellow-seeded families in direct crosses, cytoplasmic effect, brown-seeded families in reciprocal crosses, and progenies versus parents exhibited the minimum values. In contrast, yellow versus brown-seeded progenies in direct and reciprocal crosses had the maximum values.

For STI, MP, and GMP indices, crosses versus parents' effect was the highest, while brown-seeded families' effect in reciprocal and direct crosses was the lowest in value. The effect of yellow versus brown-seeded families in direct and reciprocal crosses and progenies' effect versus parents had the highest SSI index. The effect of yellow-seeded families in direct and reciprocal crosses had the lowest SSI index (Table 3).

**Table 3:** Analysis of variance (ANOVA) for tolerance indices in flaxseed families.

S.O.V	df	Mean of squares				
		TOL	MP	GMP	SSI	STI
Block	2	872.9^ns^	112.11^ns^	113.9^ns^	0.004^ns^	0.017^ns^
Family	103	7213.76**	2469.8**	2809.9**	0.71**	0.122**
Parent	7	3102.04^ns^	233.7^ns^	145.38^ns^	0.41**	0.005^ns^
Direct cross	47	6372.4**	2907.6**	3296.1**	0.77**	0.13**
Yellow	20	4270.4**	4169.4**	4610.5**	0.38**	0.16**
Brown	26	6297.1**	1838.25**	1957**	0.81**	0.10**
Yellow vs. Brown	1	29,646.7**	2965.8**	5708.7**	3.57**	0.25**
Reciprocal cross	47	7913.2**	1997**	2555.5**	0.68**	0.11**
Yellow	20	7225.8**	2485.5**	3154.8**	0.39**	0.12**
Brown	26	5912.5**	1645**	1902.8**	0.7**	0.10**
Yellow vs. Brown	1	736,669**	1378.7ns	7548.7**	5.8**	0.31**
Direct vs. Reciprocal	1	4609.5*	8978^ns^	903.8^ns^	0.025^ns^	0.03^ns^
Cross vs. Parent	1	5649.7*	12,402.3**	15,723.5**	1.41**	0.62**
Error	206	961.55	377.7	342.8	0.099	0.018
CV (%)		29	11.31	11.37	28.5	23.19

TOL: Tolerance index, MP: Mean Productivity index, GMP: Geometric Mean of Productivity index, SSI: Stress Susceptibility Index, STI: Stress Tolerance Index.

^ns^Non significant.

*Significant at 0.05 level of probability.

**Significant at 0.01 level of probability.

#### Genetic parameters

Estimates of genetic parameters such as genotypic and phenotypic coefficients of variation (GCV and PCV, respectively), broad-sense heritability (h^2^_b_) for different traits, tolerance indices, genetic advance (GA) for seed yield in the families, and Chi-square (χ^2^) values are presented in Tables 4, 5 and 6.

#### Means and coefficients of variation

In the non-stress condition, means of all traits were higher for yellow-seeded families compared to the brown-seeded ones except for NC (in BS (brown seeded) (34.6), in YS (yellow seeded) (31.68)) and NB (BS (2.37), YS (2.45)), but in the water stress condition, means in brown-seeded families were higher than yellow-seeded ones for PH (BS (54.52), YS (53.97)), NB (BS (2.7), YS (2.4)), NC (BS (19.74), YS (17.02)), NSC (BS (7.18), YS (6.9)), TWS (BS (4.6), YS (4.41)), and seed yield (BS (138.5), YS (107.2)). In this condition, means of brown-seeded families were higher for MP (BS (169.47), YS (162.47)), GMP (BS (160.88), YS (150.6)), and STI (BS (0.57), YS (0.51)) than yellow-seeded ones, and for TOL (BS (89.22), YS (110.43)) and SSI (BS (0.95), YS (1.21)) indices, they were lower in brown-seeded families (Table 4).

In the non-stress environment, the highest and the lowest GCV were observed for NB (22.04) in brown-seeded families and NSC (1.12) in yellow-seeded families, respectively. In contrast, in the water stress condition, the highest GCV was related to seed yield (33.21) in yellow-seeded families, and the lowest one was observed for DC (1.8) in brown-seeded families (Table 4). For drought tolerance indices, the highest value of GCV was for the SSI index in brown-seeded families (50.52), and the lowest value belonged to the MP index in brown-seeded families (13.01).

In general, in the non-stress environment, GCV in brown-seeded families was more than that in yellow-seeded ones for all the traits except seed yield. Still, it was lower in brown-seeded families in the water stress environment than in yellow-seeded ones for DC and NC traits. The PCV parameter in the non-stress environment had the highest value for NB (44.08) in brown-seeded families, and the lowest value was for DC (5.1) in yellow-seeded families (Table 4). In the stress environment, the highest PCV value belonged to seed yield (61.05), and the lowest one was for DC (3.22) in yellow-seeded families. Also, for drought tolerance indices, the highest PCV was for the SSI index (94.73), and the lowest one belonged to the MP index (25.29) in brown-seeded families.

**Table 4:** Mean, genotypic coefficient of variation, phenotypic coefficient of variation and heritability for morpho-phenological traits and tolerance indices of yellow (Y) and brown (B) flaxseed families under normal and stress conditions

Mean						PCV				GCV				h_b_^2^ (%)			
Env	Normal		Stress		LSD (0.05)	Normal		Stress		Normal		Stress		Normal		Stress	
Trait	Y	B	Y	B		Y	B	Y	B	Y	B	Y	B	Y	B	Y	B
DF	62.84 ± 0.35^a^	62.4 ± 0.39^ab^	62.14 ± 0.32^b^	61.69 ± 0.3^c^	2.58	6.46	7.53	5.74	5.93	3.37	4.04	3.1	3.2	0.82	0.86	0.87	0.88
DC	69 ± 0.36^a^	68.66 ± 0.32^ab^	67.9 ± 0.25^b^	67.62 ± 0.2^c^	2.12	5.1	5.31	3.22	3.4	2.68	2.79	2.01	1.8	0.82	0.83	0.80	0.81
DR	97.68 ± 0.42^a^	97.12 ± 0.37^a^	86.2 ± 0.42^b^	86.01 ± 0.36^b^	2.11	8.2	5.38	6.56	6.68	3.24	3.07	3.84	3.8	0.97	0.97	0.98	0.98
PH	66.78 ± 0.93^a^	63.44 ± 1.29^b^	53.97 ± 0.88^d^	54.52 ± 0.99^c^	8.12	14.48	17.88	18.6	21.68	7.20	15.16	9.54	11.5	0.74	0.91	0.78	0.84
NB	2.37 ± 0.09^c^	2.45 ± 0.08^b^	2.4 ± 0.07^b^	2.7 ± 0.06^a^	0.92	37.97	44.08	30.83	32.22	17.72	22.04	12.25	14.1	0.62	0.73	0.48	0.59
NC	31.68 ± 1.1^b^	34.6 ± 1.06^a^	17.02 ± 0.47^d^	19.74 ± 0.41^c^	11.2	34.56	42.54	29.61	24.51	9.82	18.72	10.57	9.22	0.24	0.48	0.39	0.34
CD	6.79 ± 0.04^a^	6.77 ± 0.03^a^	6.59 ± 0.04^b^	6.58 ± 0.04^b^	0.31	7.36	7.68	9.1	8.2	3.83	3.84	4.70	4.7	0.76	0.76	0.77	0.70
NSC	8.87 ± 0.07^a^	8.87 ± 0.06^a^	6.9 ± 0.08^c^	7.18 ± 0.06^b^	0.95	7.66	8.79	11.3	12.67	1.12	2.7	2.9	4.6	0.53	0.33	0.19	0.39
TWS	5.12 ± 0.11^a^	5.15 ± 0.14^a^	4.41 ± 0.09^c^	4.6 ± 0.1^b^	0.22	26.17	31.84	25.85	30.65	14.65	18.06	14.51	17.4	0.94	0.96	0.93	0.94
Yield	217.6 ± 5.83^a^	211.2 ± 4.34^b^	107.2 ± 4.81^d^	138.5 ± 4.2^c^	63.4	40.28	29.27	61.05	38.82	18.92	14.25	33.21	20.9	0.82	0.71	0.88	0.83
Mean						PCV				GCV				h_b_^2^ (%)			

Seed color	Y	B	LSD (0.05)	Y	B	Y	B	Y	B								
TOL	110.43 ± 5.15^a^	89.22 ± 3.94^b^	70.1	59.16	88.93	30.07	47.26	0.33	0.54								
MP	162.47 ± 3.36^b^	169.47 ± 2.17^a^	31.23	39.74	25.29	21.88	13.01	0.31	0.39								
GMP	150.6 ± 3.47^b^	160.88 ± 2.24^a^	29.77	45.08	27.49	25.03	14.41	0.31	0.78								
SSI	1.21 ± 0.04^a^	0.95 ± 0.04^b^	0.40	50.41	94.73	25.62	50.52	0.62	0.24								
STI	0.51 ± 0.022^b^	0.57 ± 0.016^a^	0.22	78.43	54.38	43.13	28.94	0.58	0.66								

Different letters (a–d) within the columns indicate significant difference at *P* < 0.05.

*DF* Day to flower, *DC* day to capsule, *DR* day to ripening, *PH* plant height, *NB* number of branches. *NC* number of capsule, *CD* capsule diameter, *NSC* number of seed in capsule, *TWS* thousand weight of seeds, *TOL* tolerance Index, *MP* mean productivity Index, *GMP* geometric mean of productivity Index, *SSI* stress susceptibility index, *STI* stress tolerance index. *Mean* average over all families, *PCV* phenotypic coefficient of variation, *GCV* genotypic coefficient of variation, *h*_*b*_^2^ broad-sense heritability.

#### Heritability, genetic advance, and Chi-square test

Broad-sense heritability was higher than 50% for most morpho-phenological traits except for the number of capsules per plant and the number of seeds per capsule in both brown and yellow seeded families at both moisture environments and the number of branches per plant in brown-seeded families in the water stress environment. High heritabilities were obtained for days to ripening (in stress: 0.98; in non-stress: 0.97), thousand seeds weight (stress: 0.94; non-stress: 0.96), days to flowering (stress: 0.88; non-stress: 0.86) and seed yield (stress: 0.82; non-stress: 0.88) (Table 4).

In the present study, the genetic advance (GA) for seed yield in brown progenies of crosses was higher than yellow-seeded families except in some cases. For most progenies, higher levels of GA were observed in non-stress conditions for both seed color families. Only in some cases, more genetic advances were observed in water stress conditions, e.g., progenies from the crosses of parents 1, 8, and 5 (Table 5). The highest estimation of genetic advance was observed in brown and yellow-seeded families where either genotype #3 or #7 were one of the parental lines in the crosses, e.g., 3 × 6b (116.81), 3 × 7b (113.36), 3 × 7y (126.92),7 × 5y (171.35), and 7 × 6y (149.70) (Table 5).

The results obtained from the Chi-square analysis in non-stress conditions showed that the cytoplasmic effect was significant in brown-seeded families for DR (35.87), PH (36.4), NB (46.72), NC (37.91), CD (50.71), and seed yield (33.26) (Table 6). In yellow-seeded families, there was no significant cytoplasmic effect for the measured traits except for CD (37.43) (Table 6). Under the water stress condition, the cytoplasmic effect was significant in brown-seeded families for DR (59), NB (49.37), NSC (46.27), and TWS (37.43). In yellow-seeded families, it was significant only for DR (50.72) and seed yield (28.95) (Table 6).

**Table 5:** Genetic advance (GA) for seed yield in brown and yellow-seeded families at two moisture environments.

Parent ♂	1		2		3		4		5		6		7		8	
Parent♀	Normal	Stress	Normal	Stress	Normal	Stress	Normal	Stress	Normal	Stress	Normal	Stress	Normal	Stress	Normal	Stress
**Brown**																
1	−	−	19.57	− 3.73	75.06	− 27.10	16.81	− 30.32	59.54	37.32	74.77	76.43	22.07	18.95	− 10.31	− 3.03
2	40.84	29.90	−	−	20.64	− 6.47	68.98	9.85	49.38	13.25	–	–	63.88	− 2.12	82.77	− 1.10
3	90.94	25.63	19.87	− 9.63	−	−	66.18	12.75	84.85	39.52	45.82	61.03	113.36	− 4.98	83.71	5.97
4	22.67	− 15.48	59.13	− 22.25	44.60	− 25.52	−	−	4.27	32.20	74.03	52.32	44.02	30.77	36.68	44.98
5	− 57.26	66.25	62.25	9.25	–	–	12.07	37.80	−	−	79.51	37.95	55.53	82.47	74.08	26.08
6	7.01	35.97	56.62	6.83	116.81	48.30	60.37	43.38	100.10	11.62	−	−	28.39	− 33.42	− 41.93	40.67
7	− 6.08	− 33.32	30.65	− 30.12	68.92	56.02	21.93	28.83	33.80	16.33	59.57	− 25.62	−	−	37.49	30.92
8	− 28.83	19.83	30.37	22.83	61.65	43.97	− 5.12	67.38	111.08	43.28	25.12	36.00	35.82	− 25.95	−	−
**Yellow**																
1	−	−	−	−	−	−	−	−	− 2.26	− 2.42	− 6.70	− 22.03	75.21	− 37.12	62.89	40.97
2	−	−	−	−	−	−	−	−	− 30.27	− 14.88	–	–	83.65	− 16.45	61.00	45.50
3	−	−	−	−	−	−	−	−	32.92	− 28.22	43.62	− 15.03	126.92	− 2.92	132.43	1.17
4	−	−	−	−	−	−	−	−	48.20	− 35.20	64.37	6.45	52.53	− 18.30	50.82	− 38.68
5	26.21	− 35.95	0.64	− 31.15	−	−	− 13.53	− 26.40	−	−	102.72	− 20.12	171.35	− 27.27	47.22	38.75
6	37.91	2.63	− 21.43	− 90.83	92.82	− 54.37	0.43	− 64.02	− 35.30	− 91.58	−	−	99.70	14.05	63.72	− 100.60
7	31.67	− 4.65	22.63	− 34.78	141.51	− 3.65	65.40	− 17.30	89.87	9.27	149.70	− 18.28	−	−	137.82	21.05
8	19.4	52.97	41.51	18.83	112.97	60.63	16.35	− 10.35	30.22	40.22	95.32	− 17.00	98.48	− 3.28	−	−

♂: Male parents, ♀: Female parents.

**Table 6:** Chi-square test for the cytoplasmic effect based on genetic variation.

Moisture environment	Normal condition		Stress condition	
Trait	Seed color			
	Yellow	Brown	Yellow	Brown
DF(day)	18.9^ns^	23.74^ns^	16.53^ns^	22.43^ns^
DC (day)	11.68^ns^	15.63^ns^	9.76^ns^	14.47^ns^
DR (day)	30.54^ns^	35.87*	50.72**	59**
PH (cm)	10.6^ns^	36.4*	16.5^ns^	24.38^ns^
NB(pp)	14.11^ns^	46.72**	13.54^ns^	49.37**
NC(pp)	14.45^ns^	37.91**	18.7^ns^	16.85^ns^
CD (mm)	37.43**	50.71**	27.96^ns^	25.35^ns^
NSC(pp)	14.03^ns^	20.64^ns^	10.47^ns^	46.27**
TWS (g)	16.6^ns^	26.88^ns^	18.46^ns^	37.43**
Seed yield (g/m^2^)	22.01^ns^	33.26*	28.95*	23.95^ns^

DF: Day to Flower. DC: Day to Capsule. DR: Day to Ripening. PH: Plant Height. NB: Number of Branches. NC: Number of Capsules. CD: Capsule Diameter. NSC: Number of Seeds in Capsule. TWS: Thousand Weight of Seeds. pp: per plant.

^ns^Non significant.

*Significant at 0.05 level of probability.

**Significant at 0.01 level of probability.

#### Multivariate data analysis

The principal component analysis was used to investigate parental diversity and the difference between yellow and brown-seeded offspring from direct and reciprocal crosses. According to Table 7, only families with both yellow and brown-seeded progenies were used to construct the biplot, and families with only brown-seeded progeny were excluded.

**Table 7:**
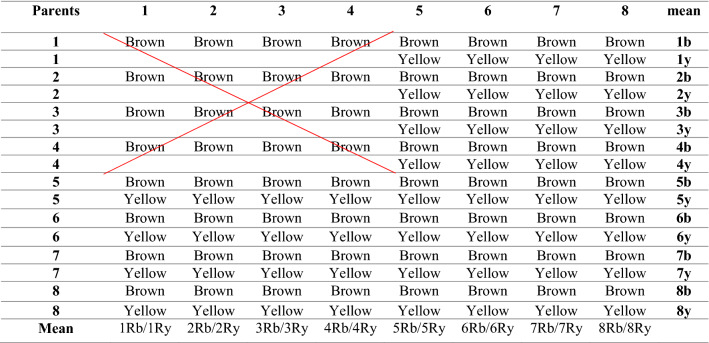
Yellow and brown-seeded families from direct and reciprocal crosses used in the biplot analysis.

Y: Yellow families, b: Brown families, Ry: Reciprocal yellow families, Rb: Reciprocal brown families.

According to the table, families with only brown offspring were eliminated from the analysis to have the same balance for the effect of yellow and brown seed colors on biplot analysis.

**Figure 1:**
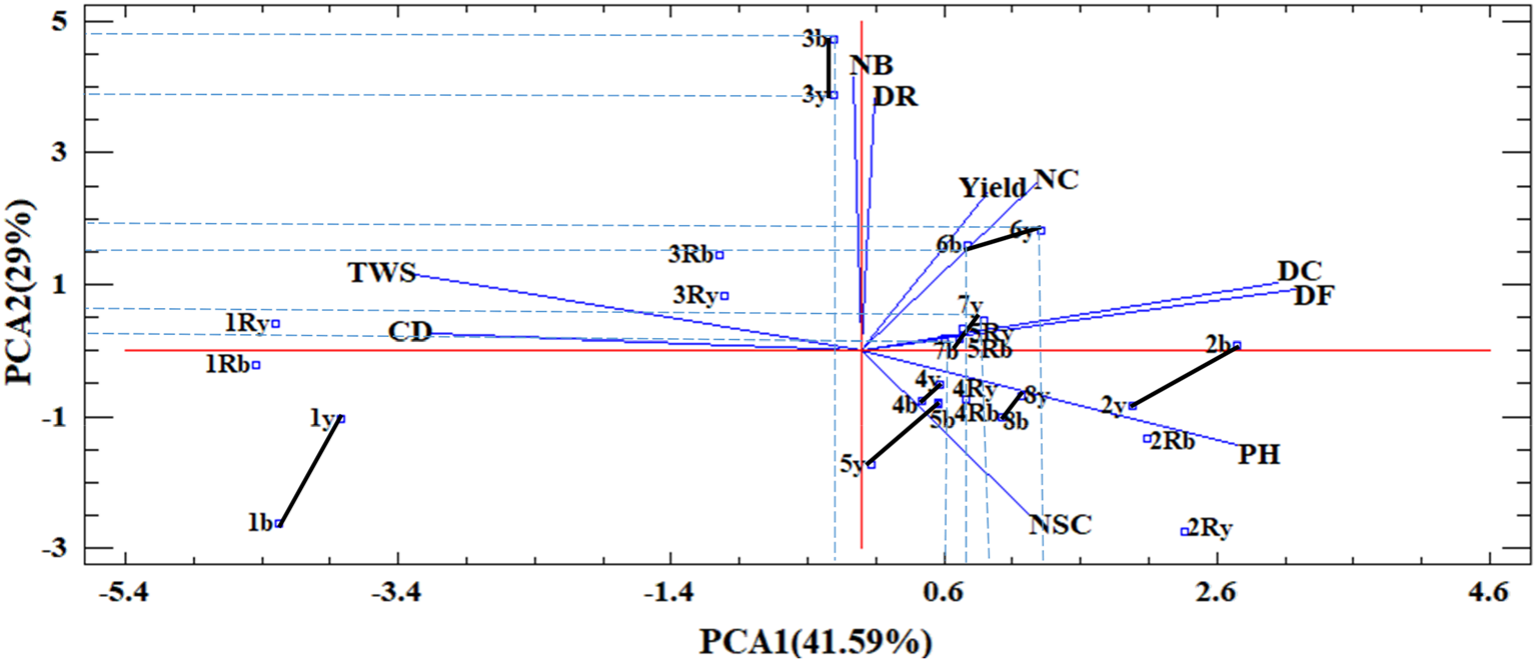
The biplot displaying contribution of different traits in the variability of yellow and brown-seeded families from direct and reciprocal crosses of flax under non-stress condition. DF: Day to Flower. DC: Day to Capsule. DR: Day to Ripening. PH: Plant Height. NB: Number of Branches. NC: Number of Capsule. CD: Capsule Diameter. NSC: Number of Seeds in Capsule. TWS: Thousand Weight of Seeds. Yield: seed yield. y: Yellow, b: Brown, Ry: Reciprocal yellow, Rb: Reciprocal brown. Solid lines indicate the distance between two seed color groups in each parent in the space of PCs.

The first two components explained 41.59 and 29% of the total variance in the non-stress environment, 34.27 and 21% in the water stress environment, 49.44 and 27.14% in both moisture environments. Under the non-stress environment, the first principal component (PC1) positively correlated with CD and TWS and negatively correlated with DF, PH, and DC (Fig. 1). The second component (PC2) positively correlates with NC, NB, DR, seed yield, and had a negative correlation with NSC. Therefore, the number of capsule (NC) was the main component of seed yield under the non-stress environment and the number of seeds in capsule (NSC) was not correlated with yield. The selection of genotypes with low (near zero) PC1 and high-positive PC2 would increase seed productivity and would develop earliness. In this respect, families 3b, 3y, 6b, 6y, 7y, and 7b were considered superior families.

**Figure 2:**
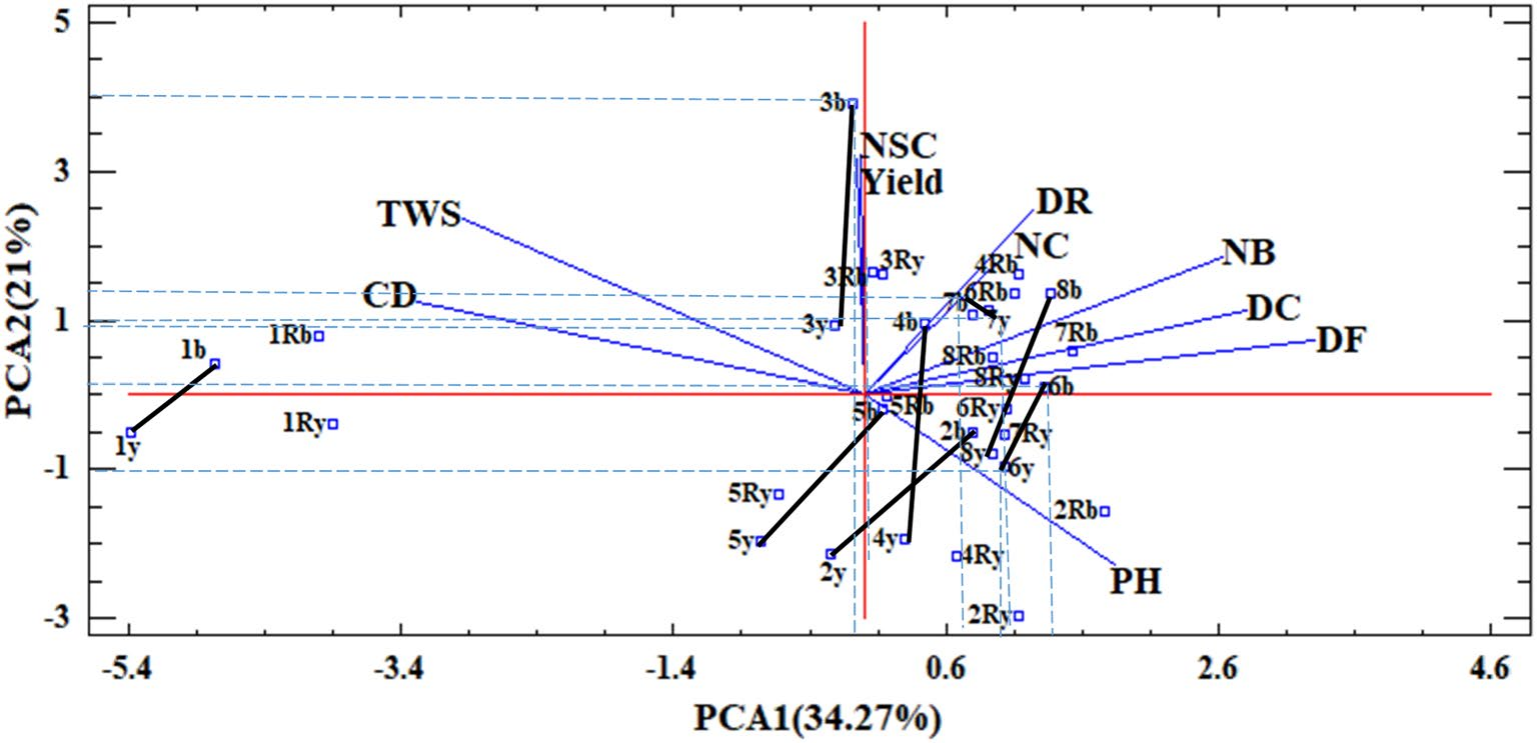
The biplot displaying contribution of different traits in the variability of yellow and brown families from direct and reciprocal crosses of flax under stress conditions. DF: Day to Flower. DC: Day to Capsule. DR: Day to Ripening. PH: Plant Height. NB: Number of Branches. NC: Number of Capsule. CD: Capsule Diameter. NSC: Number of Seed in Capsule. TWS: Thousand Weight of Seeds. Yield: seed yield. y: Yellow, b: Brown, Ry: Reciprocal yellow, Rb: Reciprocal brown. Solid lines indicate the distance between two seed color groups in each parent in the space of PCs.

Under the water stress environment, the first principal component (PC1) positively correlated with DC, DF, NB, and PH and had a negative correlation with CD and TWS (Fig. 2). The second principal component (PC2) had a positive correlation with seed yield, NSC, DR, TWS, NC, and negative correlation with PH; therefore, it was considered as a 'seed productivity component' under both tested environments. Also, as Fig. 2 shows, in contrast to the non-stress environment, the main component that influenced seed yield under water stress conditions was the number of seeds in capsule (NSC), and then by a lesser extent, day to ripening, number of capsules, thousand weight of seeds and capsule diameter, respectively. The selection of genotypes with low (near zero) PC1 and high-positive PC2 can improve seed productivity of flax and develop earliness like in the non-stress environment. In this respect, families 3b, 3y, 4b, 7b, 7y, and 8b were the superior ones.

**Figure 3:**
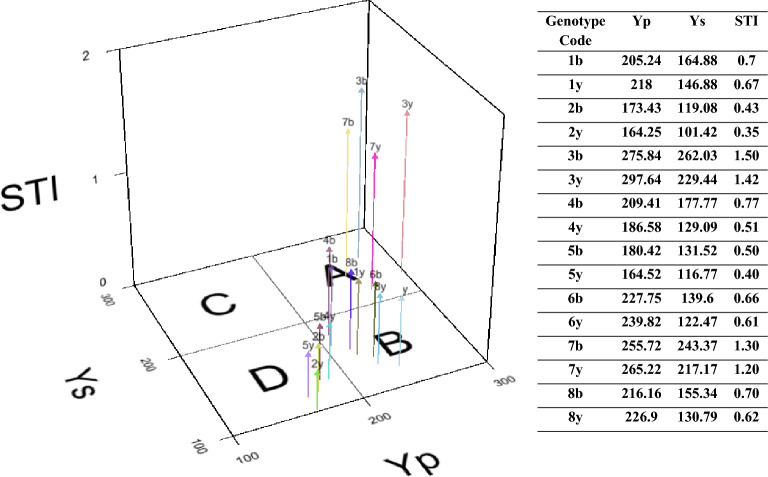
Three-dimensional plot based on Fernandez (^20^,^49^ and adjusted values of seed yield (Y_p_ and Y_s_) and the stress tolerance index (STI) obtained for yellow and brown-seeded families investigated under stress and non-stress conditions. According to this three-dimensional plot, 16 families investigated were classified into four groups A, B, C, and D. Group A included genotypes 3y, 3b, 7y, and 7b; group B consisted of families 4b, 4y, 1b, 1y, 6b, 6y, 8b, and 8y; group D included families 2y, 2b, 5y, and 5b. Group C had no family.

Figure 3 estimated STI for yellow and brown-seeded families investigated using seed yield as a screening indicator for water stress-tolerant families. Accordingly, genotypes with STI ≥ 1 were considered stress-resistant^20^. Of the 16 yellow and brown-seeded tolerant families, four of them had an STI ≥ 1, and the highest STI values were recorded for 3b (1.50), 3y (1.42), 7b (1.30), and 7y (1.20) (Fig. 3). Based on Fernandez’s theory^20^, we used a three-dimensional plot to categorize these 16 families into four groups. According to Fig. 3, families 3b (N: 275.84; S: 262.03), 3y (N: 297.64; S: 229.44), 7b (N: 255.72; S: 243.37), and 7y (N: 265.22; S: 217.17) with the highest seed yield under both moisture conditions (water stress and non-stress) were identified as water stress-tolerant families (Fig. 3).

#### Intensification of differential reaction by drought stress between seed color groups of genotypes

The parents' coordinations were calculated based on the biplot diagram and principal components to estimate water stress's effect on the intensification of the differences between seed color groups. The dashed lines on the biplot chart (Figs. 1, 2) show each point's length and width, used to calculate the differential values between two seed color groups under non-stress and water stress conditions in Table 8. Positive and higher differential values under water stress indicate that the difference between brown and yellow color groups has been intensified by water stress in one PC or both PC directions.

**Table 8:** Determination of yellow and brown families’ coordinates based on the biplot analysis of parents in two humidity environments and determination of drought stress response intensity.

Non-stress	Stress
1b: − 4 PC1; − 2.7 PC2 1y: − 3.6 PC1; − 1.1 PC2 Differential 1b-1y = (− 4 + 3.6) and (− 2.7 + 1.1) = (− 0.4PC1;− 1.6PC2)	1b: − 4.8 PC1; 0.4 PC2 1y: − 5.4 PC1; − 0.5 PC2 Differential 1b-1y = (− 4.8 + 5.4) and (0.4 + 0.5) = (0.6PC1;0.9PC2)
2b: 2.8 PC1; 0.1 PC2 2y: 2.1 PC1; − 0.5 PC2 Differential 2b-2y = (2.8–2.1) and (0.1 + 0.5) = (0.7PC1;0.6PC2)	2b: 0.8 PC1; − 0.5PC2 2y: − 0.3 PC1; − 2.2 PC2 Differential 2b-2y = (0.8 + 0.3) and (− 0.5 + 2.2) = (1.1PC1;1.7PC2)
3b: − 0.3 PC1; 4.6 PC2 3y: − 0.33PC1; 3.9PC2 Differential:3b-3y = (− 0.3 + 0.33) and (4.6–3.9) = (0.03PC1;0.7PC2)	3b: − 0.2PC1; 3.9PC2 3y: − 0.4PC1; 1PC2 Differential 3b-3y = (− 0.2 + 0.4) and (3.9–1) = (0.2 PC1;2.9 PC2)
4b: 0.4 PC1; − 1 PC2 4y: 0.6 PC1; − 0.5 PC2 Differential 4b-4y = (0.4–0.6) and (− 1 + 0.5) = (− 0.2PC1;0.5PC2)	4b: 0.5 PC1; 0.9 PC2 4y: 0.2 PC; − 2 PC2 Differential 4b-4y = (0.5–0.2) and (0.9 + 2) = (0.3PC1;2.9PC2)
5b: 0.6 PC1; − 1 PC2 5y: 0.1 PC1; − 1.6 PC2 Differential 5b-5y = (0.6–0.1) and (− 1 + 1.6) = (0.5PC1;0.6PC2)	5b: 0 PC1; − 0.1 PC2 5y: − 0.5 PC1; − 2.2 PC2 Differential 5b-5y = (0 + 0.5) and (− 0.1 + 2.2) = (0.5PC1;2.1PC2)
6b: 0.8 PC1; 1.5 PC2 6y: 1.3 PC1; 1.9 PC2 Differential 6b-6y = (0.8–1.3) and (1.5–1.9) = (− 0.5 PC1; − 0.4PC2)	6b: 1.5 PC1; 0.1 PC2 6y: 1.1 PC1; − 1 PC2 Differential 6b-6y = (1.5–1.1) and (0.1 + 1) = (0.4 PC1; 1.1 PC2)
7b: 0.63 PC1; 0.1 PC2 7y: 1 PC1; 0.6 PC2 Differential7b-7y = (0.63–1) and (0.1–0.6) = (− 0.37 PC1; − 0.5 PC2)	7b: 0.8 PC1; 1.4 PC2 7y: 1 PC1;0.9 PC2 Differential 7b-7y = (0.8–1) and (1.4–0.9) = (− 0.2 PC1; 0.5 PC2)
8b: 1.1 PC1; − 1 PC2 8y: 1.3 PC1; − 0.5 PC2 Differential 8b-8y = (1.1–1.3) and (−1 + 0.5) = (− 0.2PC1; − 0.5PC2)	8b: 1.5 PC1; 1.5 PC2 8y: 1 PC1; − 1 PC2 Differential 8b-8y = (1.5–1) and (1.5 + 1) = (0.5PC1; 2.5PC2)

y: yellow. b:brown. PC1: principle component 1, PC2: principle component 2, (based on Figs. 1, 2).

The results (Table 8) show that water stress increased the favorability of brown-seeded offspring over yellow-seeded ones for all parents. This differential intensification is especially evident for PC2, which is the seed productivity component. For parent #1, water stress has inverted the favorability of yellow-seeded progenies to brown-seeded ones. The highest intensification by water stress between the two seed color groups was found for parents # 3, 4, and #8 in the PC2 direction, and the lowest intensification was found for parent # 7 (Table 8).

#### Relations between lignans biosynthesis and drought stress adaptation

**Figure 4:**
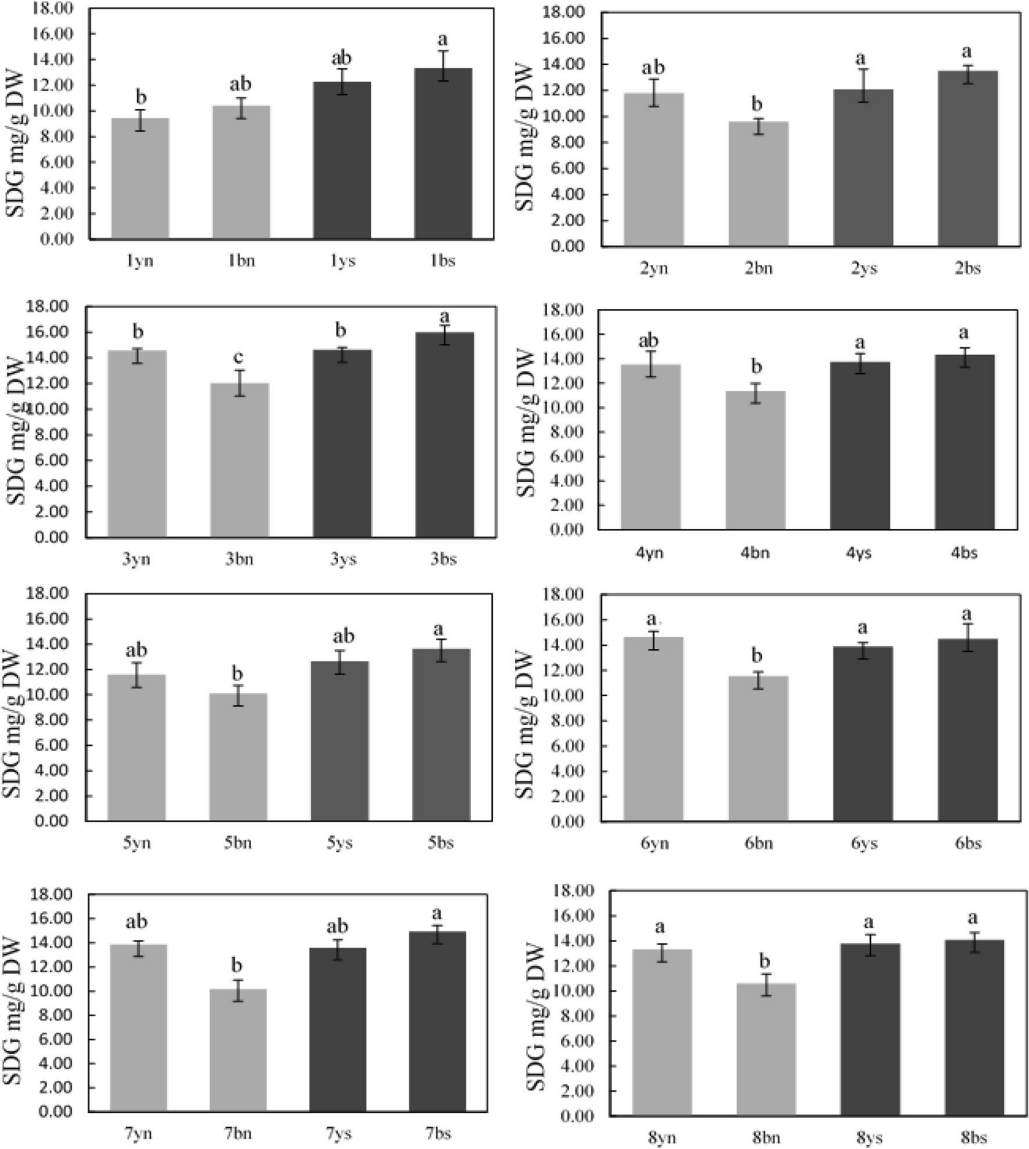
Comparison of SDG content (secoisolariciresinol diglucoside, in mg g^−1^ dry weight) extracted from families obtained from the diallel cross of flax under water stress and normal irrigation conditions. In each population’s diagram, the same letter above columns indicates that values are not statistically different (*p* > 0.05). Values are mean ± standard error. Letters y, b, n and s denote yellow color, brown color, normal conditions and stress conditions, respectively.

According to the Fernandez diagram (Fig. 3), families #7 and #3 especially brown-seeded ones had the highest seed yield under water stress and normal conditions, and the lowest seed yield was observed in families #2 and #5. The amount of SDG lignans was also evaluated in all families (Fig. 4). Comparing the mean of lignans (SDG) content in populations obtained from crossing in the non-stress environment showed that the highest amount of SDG was in yellow-seeded families #3 (14.76 mg g^−1^DW), #6 (14.64 mg g^−1^DW), and #7 (13.86 mg g^−1^DW) and the lowest amount of lignans was in brown-seeded families #2 (9.6 mg g^−1^DW) and #5 (10.11 mg g^−1^DW). Interestingly, the amount of SDG increased due to water stress in most families and this intensification was higher in brown seeds so that the highest amount of SDG in the water stress environment was observed in families #3 (16 mg g^−1^DW) and #7 (14.93 mg g^−1^DW), those with brown seeds and the highest seed yield under water stress conditions, and the lowest amount was in families #2 (12.08 mg g^−1^DW), #1 (12.25 mg g^−1^DW) and #5 (12.66 mg g^−1^DW) with yellow seeds (Fig. 4).

## Discussion

In plant breeding, accurate information about the relationship between yield and yield components greatly facilitates selection to improve yield. In our study, the relation between seed coat color and flax seed yield, yield components, and the amount of secondary metabolites such as lignans is evident, which is also intensified by water stress. The relationship between the seed color and other agronomic traits and the effect of seed color on qualitative characteristics such as oil content, fatty acid percentage, phenylpropanoids content, and protein content has been previously reported in different plants such as common vetch, rapeseed, safflower, and also flaxseed^13,21–24^. Our findings using multiple full-sib families of flax each pair sharing common genetic background but different in seed colors confirm these associations likewise, for instance in traits such as seed yield, TWS, NSC, NC, etc.

The analysis of variance showed that under non-stress conditions, the difference between yellow and brown-seeded families was not significant on both direct and reciprocal crosses for all of the studied traits, especially the seed yield. However, under water stress conditions, this effect was significant for most of the measured traits including the seed yield. This was also evident from mean comparisons (Table 4), which showed water stress reduced the value of most of the traits, including seed yield and yield components in yellow-seeded families more than the brown-seeded ones. Saeidi and Rowland^11,12,25^, Sood et al.^18^, and Soto Cerda et al.^26^, also reported that flaxseed color can be highly correlated with seed yield-related traits. Indeed, the higher seed SDG content and possibly other secondary metabolites in the brown-seeded families may explain their higher stability under stress conditions. The comparison of means for phenological traits, including DF, DC, and DR showed that brown-seeded families had a faster growth rate (earliness) than yellow-seeded ones. This may have helped them escape from stress conditions and also had higher seed yield components including NSC and TWS which positively influence the seed yield in brown-seeded families under stress conditions.

Stress tolerance indices have been extensively used in different crops to identify high-seed-yield genotypes under normal and drought stress conditions^26–28^. In this study, brown-seeded families showed higher MP, GMP, and STI indices and lower TOL index than yellow-seeded ones, indicating that brown-seeded families were more stable under stress conditions in terms of productivity. Higher water stress tolerance in brown-seeded families may be attributed to secondary metabolites biosynthesis such as lignans and tannins in the seed coat. Similar to this interpretation, Asgarinia et al.^27^, Pizzi and Cameron^29^ and Hassanpour et al.^30^ reported that brown-seeded genotypes were more resistant to drought stress due to the higher hardiness of seed coat, the presence of secondary metabolites, tannins, and their antioxidant properties compared to yellow-seeded genotypes. Tannins present in the brown seed coat can also influence seed vigor^34^. In addition, lignans and flavonoids may also act as hormone-like compounds, for UV protection, and as defensive compounds against herbivores and pathogens^30,31^. Therefore, increased lignan biosynthesis due to water stress in brown-seeded families can be another factor in their greater resistance to stress^32,33^. Drought per se may increase the content of tannins and lignans in plants and seeds, and simultaneously, the effect of tannins and lignans on resistance under a stress environment increases^29^.

Considerable differences were observed between GCV and PCV for seed yield and NSC at both moisture environments for yellow-seeded lines when compared to their brown-seeded counterparts. This may indicate that observed variations were mostly due to the environmental influence over these characters in the yellow-seeded group and families with brown seeds have higher stability of seed yield and yield components in different moisture environments. These results are consistent with the previous investigations reported in the literature^11,12,34^. One of the main reasons behind this yield stability in brown-seeded genotype may be attributed to secondary metabolites biosynthesis, especially lignans and tannins. It has been reported that the higher amount of anthocyanins and proanthocyanidins in dark-coat-color seeds, make them more resistant to different environmental conditions^24,35,36^.

A higher heritability estimate for a particular trait indicates the greater role of genetic factors in controlling that trait and the possibility of its improvement by an appropriate selection program^37^. Broad-sense heritability for most of the studied traits except for NSC and NB was higher under water stress than in the non-stress environment. Also, heritabilities of all traits were higher in brown-seeded families than the yellow-seeded ones at water stress conditions. Under the non-stress condition, heritabilities were higher in yellow-seeded families except for NSC and seed yield. These findings are in agreement with the results of Saeidi^34^, reporting that traits’ values of DF, DC, DR, and TWS in flax families with brown seeds were higher than those in the yellow-seeded genotypes.

The highest amount of GA was observed in direct and reciprocal crosses in non-stress conditions for parents # 3 and 7, yellow in the seed. In contrast, in water stress conditions, high levels of genetic advance were observed in brown-seeded progenies of all parents, especially # 7 and # 4. Given that no significant cytoplasmic effect was observed for seed yield, the amount of GA in progenies of direct and reciprocal crosses was almost the same.

The three-dimensional plot based on seed yield under non-stress (Yp) and water stress (Ys) conditions and stress tolerance index (STI) divided yellow and brown-seeded families into three groups (Fig. 3). This indicated that under water stress situations, the difference between yellow and brown-seeded families was more significant. Fernandez^20^ classified plant genotypes into four groups: A, B, C, and D based on seed yield in stress and non-stress environments. Genotypes in groups A and D contrast each other so that those in group A yield well in both stress and non-stress environments but the ones in group D yield poorly in both situations. Similarly, genotypes of groups B and C oppose each other as those of B yield well only under a non-stress environment, and the ones in group C yield relatively well only under stress environments. According to Fernandez, the most appropriate index for stress tolerance is STI that could distinguish the first group (A) from the other groups^20^. In our study, families 3b, 3y, 7b, and 7y were characterized in group A (Fig. 3). According to our results, the STI index was higher for all the brown-seeded families than the yellow ones, indicating the higher yield stability of brown-seeded families under water stress conditions, which is consistent with the findings of other studies^11,24,34^. As a result, it was shown that family # 3, followed by # 7, had higher performance in both normal and water stress conditions.

High seed yield in brown families under water stress can also be due to the intensification of lignans biosynthesis in their seed coat other than tannins, anthocyanins, and proanthocyanidins. According to Fig. 4, under normal conditions, the amount of SDG was higher in families with yellow seeds than that of brown seeds. Under water stress conditions, the amount of SDG increased in most offspring; however, the increase in lignans (SDG) content was higher in families with brown seeds than those with yellow seeds. Therefore, in contrast to normal water conditions, brown seeds had significantly higher SDG content in water stress conditions. Lignans have a very important role in plant development i.e., interactions and adaptations to ever-changing environments^38,39^. Kirakosyan et al.^40^ and Rezayian et al.^41^ reported that the amount of polyphenols in various plants, such as *Crataegus laevigata* and *Brassica napus*, has increased under conditions of water stress and other abiotic stresses. As lignans (SDG) are types of polyphenols; therefore, increasing the amount of lignans in brown seed families may increase drought tolerance and their grain yield under water stress conditions as well.

In general, seed coat color is one of the critical characteristics, which is used to determine both the quality and commercial value of the seed. This factor can help select the appropriate seed in breeding programs and investigate the relationship between seed coat color, seed yield, yield components, and lignans, leading to the development of suitable cultivars.

Using multiple full-sib families of flax each pair sharing common genetic background but different in seed colors it was shown that the highest mean of seed yield was obtained in yellow-seeded families under normal water conditions. However, under water stress conditions, higher seed yield was observed in brown-seeded families. This may suggest that yellow-seeded varieties may be preferred for normal water conditions, but under a water stress environment, brown-seeded varieties are superior. Also, the lower difference between GCV and PCV for seed yield of brown-seeded families indicated more production stability of brown-seeded genotypes in different environmental conditions. This was also confirmed by the calculated drought tolerance indices, including MP, GMP, and STI, in which higher values were observed for brown-seeded families. In the present study, heritability and genetic advances were higher for brown-seeded families than the yellow ones at water stress conditions. Also, at normal water conditions, the highest amount of SDG was observed in yellow-seeded families but under the water stress environment it was the opposite and a higher amount was observed for the brown-seeded families. This may suggest different breeding strategies under water stress and non-stress conditions and for SDG content in respect to seed color. Water stress also intensified different responses of the two seed color categories regarding seed yield, the amount of SDG, and agro-morphological traits. Plant height, seed yield, days to ripening, and the number of capsules per plant were reduced to a greater extent in yellow-seeded families than the brown ones under water stress conditions. This may have considerable consequences when breeding flax for drought environments.

## Material and methods

### Plant material, experimental site, and design

The plant material for this study consisted of F_3_ progenies from an 8 × 8 diallel cross designed based on contrasting parental traits, especially for seed and flower colors (Table 9). The eight parental genotypes for these crosses were selected from among 144 genotypes (world collection), obtained from IPK gene bank (Germany), based on a previous comprehensive field evaluation^42^. In collection and crossing of the parental material, we complied with our relevant institutional and national standards, and also with international guidelines and legislation complied with the IUCN Policy Statement on Research Involving Species at Risk of Extinction and the Convention on the Trade in Endangered Species of Wild Fauna and Flora.

To produce full-sib lines of similar genetic background with different seed colors, all 64 possible direct and reciprocal crosses were performed for the eight selected parents. After crossing, two selfing generations were allowed, and F_3_ families segregated into brown and yellow seed colors were obtained. Direct and reciprocal crosses of brown-seeded parents produced only brown seed progenies. Still, crosses of yellow with brown and yellow with yellow resulted in progenies having both yellow and brown seed colors. The yellow and brown-seeded progenies of each F_3_ line were planted adjacently in plots in which rows were two meters in length and 25 cm apart. All F_3_ lines along their parents were field planted according to a randomized complete block design with three biological replications under two moisture environments. The experimental site was the research farm of Isfahan University of Technology located 40 km southwest of Isfahan in Lavark, Najaf Abad region (latitude 32° 32′ north and longitude 51° 22′ east), with 1630 m above sea level. For this site, the average annual temperature is 14 °C, and its annual rainfall is below 140 mm, with a specific soil density of 1.34 g cm^3^ and pH = 7.5.

**Table 9:** Parental information used in the 8 × 8 diallel cross in respect to seed and flower colors.

Parents	IPK code or name	Origin	Flower color	Seed color
1	Indian	India	White	Brown
2	LTU1474	Lithuania	White	Brown
3	KO37	Iran	Blue	Brown
4	FRA806	France	Blue	Brown
5	FRA771	France	White	Yellow
6	USA1580	United States	White	Yellow
7	SP1066	Canada	Blue	Yellow
8	Golden	Canada	Blue	Yellow

### Water stress treatment

To assess the response of brown and yellow-seeded flax sister lines to various water environments, two water treatments including normal and deficit irrigation conditions, were applied based on the maximum permissible drainage rate (MAD) of soil available water (SAW)^43^. For normal moisture conditions, plants were irrigated when 50% of SAW was drained from the root zone, and for stress treatment, irrigation was performed when 80% of SAW was depleted. Water stress treatment was applied from the late flowering stage until plant seed maturity (late April to the end of July in 2018). The irrigation intervals (days between two irrigations) were determined based on meteorological data and evapotranspiration records.

Soil moisture was measured according to standard gravitational methods^44^ at three depths of 0–20, 20–40, and 40–60 cm. The depth of irrigation was determined based on the following two equations:

$$\uptheta _{{{\text{irri}}}} =\uptheta _{{{\text{FC}}}} - \left( {\uptheta _{{{\text{FC}}}} -\uptheta _{{{\text{PWP}}}} } \right) \times {\text{MAD}} $$

where θ_irri_ is the mean soil moisture content at the root development depth at irrigation time under non-stress treatment, θ_FC_ is soil moisture content at field capacity, ρ = 1.4 g cm^−3^, θ_pwp_ is soil moisture content at the wilting point, and MAD is depletion of 50 or 80% of the total available water^43^.

$$ {\text{D}}_{{{\text{irrig}}}} = ({{\theta}}_{{{\text{fc}}}} - {{\theta}}_{{{\text{avg}}}} ) \times {\text{D}}_{{{\text{rz}}}} $$

In which D_irrig_ is the irrigation depth (cm), D_rz_ is the depth of root zone (cm), and θ_avg_ is soil–water content at the root zone before irrigation (m^3^/m^3^). Irrigation was performed using a drip system, and the volume of water used in each treatment was measured using a volumetric counter.

### Morpho-phenological traits evaluation

To examine possible response variation of genotypes in the two seed color categories, the following phenological and yield-related traits were evaluated under both irrigation regimes. Days to 50% flowering (DF), days to 50% capsule formation (DC), and days to ripening (DR) as the number of days to complete ripening. Plant height (PH), number of branches (NB) per plant, number of capsules (NC) per plant, capsule diameter (CD) and number of seeds per capsule (NSC) were measured on ten randomly selected plants in each plot after full ripening. Thousand seed weight (TWS) and seed yield were measured after harvesting.

### Analysis of flax lignans

Seeds collected from the two brown and yellow color genotypic groups, grown under normal and water stress conditions, were analyzed for lignans and SDG for the probable effects of both seed color and the water environment. The high-performance liquid chromatography (HPLC, model Agilent 1090, with diode array detection (DAD) system) was used to determine the lignans of extracts obtained from all studied families. On two samples for each family as technical replications, HPLC separation of SDG from flaxseed was performed according to the method described by Mukker et al.^45^ with some modifications. Briefly, for extraction of lignans, 250 mg of flaxseed sample was added to 2.5 ml of the extraction solvent (EtOH 75%, HPLC grade, Merck) and mixed. The samples were then placed on ultrasound (45 kHz, 50 °C) for 1 h. The extract was then allowed to cool down at room temperature for at least 30 min (or overnight in a cold room) and then neutralized (up to pH: 7) using acetic acid. The extracts were centrifuged at 5000×g for 15 min and then the supernatant was filtered through a 0.22 μm filter and the extract was prepared for injection in HPLC. The calibration curve was used to quantify the lignans and the results were calculated as mg (secoisolariciresinol diglucoside) per gram of dry weight. Calibration curves, limits of detection and quantification, as well as the validation of the method, were described by Anjum et al.^46^.

### Stress tolerance indices

Stress tolerance indices were considered to identify high-seed-yield genotypes under normal and water stress conditions. Different indices of tolerance and susceptibility to water stress were calculated based on the F3 families' performance in the normal and water stress conditions and then analyzed statistically. Fisher and Maurer^47^ proposed a stress susceptibility index (SSI) assessed using the following formula:

$$SSI=1-(Y_{s}/Y_{p})/1-({\overline{Y}_{s}}/\overline{Y_{p}})$$

For the tolerance index (TOL) and mean productivity index (MP), Rosielle and Hamblin^48^ method was followed:

$$ {\text{TOL}} = {\text{Y}}_{{\text{P}}} - {\text{Y}}_{{\text{S}}} $$

$$ {\text{MP}} = \frac{{{\text{Y}}_{{\text{P}}} + {\text{Y}}_{{\text{S}}} }}{2} $$

The geometric mean of productivity (GMP) was calculated, according to Fernandez^20^:

$$ {\text{GMP}} = \sqrt {{\text{Y}}_{{\text{P}}} \cdot \,{\text{Y}}_{{\text{S}}} } $$

Fernandez^49^ also presented the stress tolerance index (STI) based on GMP.

$$ {\text{STI}} = \frac{{{\text{Y}}_{{\text{P}}} \times {\text{Y}}_{{\text{S}}} }}{{\left( {{\overline{\text{Y}}}_{{\text{P}}} } \right)^{2} }} $$

Y_S_ was the seed yield of genotypes under stress conditions (Kg/ha). Y_P_ was the seed yield of genotypes under normal irrigation conditions, and ${\overline{\text{Y}}}_{{\text{S}}}$ and $\overline{{\text{Y}}}_{{\text{P}}}$ were the average seed yield of all genotypes under stress and normal irrigation conditions, respectively.

### Statistical analysis

#### Univariate and multivariate data analysis

After normality testing, the data were subjected to analysis of variance using the GLM method (generalized linear model) for each environment separately based on a randomized complete block design with three replications, using SAS statistical software (ver. 9.4; SAS Institute Inc. Cary, NC, USA)^50^.

Multivariate statistical analysis, including biplot analysis, was performed on the standardized data using Stat Graphics centurion XVIII (http://www.statgraphics.com). Also, to identify the genotype(s) or color groups with high productivity under both stress and non-stress conditions, a three-dimensional plot was constructed based on stress tolerance index (STI) and seed yield obtained under both water conditions using iPASTIC software^51^.

### Genetic analysis

To see which one of the two seed color groups was more influenced by environmental changes imposed by water treatment, the genetic coefficient of variation (GCV) and also the phenotypic coefficient of variation (PCV) were calculated. The GCV was calculated as:

$$ {\text{GCV}} = (\upsigma _{{\text{g}}} /\upmu ) \times 100 $$

and the PCV as:

$$ {\text{PCV}} = (\upsigma _{{\text{p}}} /\upmu ) \times 100 $$

where $\upsigma _{{\text{g}}}$ was the square root of the genotypic variance, $\upsigma _{{\text{p}}}$ was the square root of the phenotypic variance, and µ refers to the mean of the trait^52^.

To summarize how much of the variation in each trait was due to variation in genetic factors, broad-sense heritability (h_b_^2^) was calculated according to Kumar et al.^52^:

$$ {\text{h}}_{{\text{b}}}^{2} = \frac{{\updelta _{{\text{g}}}^{2} }}{{\updelta _{{\text{g}}}^{2} + \frac{{\updelta _{{\text{e}}}^{2} }}{{\text{r}}}}} $$

where $\updelta _{{\text{g}}}^{2}$ was the genotypic variance, $\updelta _{{\text{e}}}^{2}$ was the error variance, and r was the number of blocks^53,54^.

One of the essential methods to estimate the differences between parents and their offspring in self-pollinating crops is the genetic advance calculation. The following formula was used in this respect^55^:

$$ {\text{GA}} = \mu_{O} - \mu_{P} $$

where $\mu_{O}$ is the offspring mean ($\overline{F}$ 3), and $\mu_{P}$ is the parents’ mean.

Also, to evaluate the effects of cytoplasmic replacement (direct vs. reciprocal crosses), a Chi-square table was created to compare variances of the two direct and reciprocal crosses in each seed color group and two irrigation conditions.
